# Phylogenetic approaches to microbial community classification

**DOI:** 10.1186/s40168-015-0114-5

**Published:** 2015-10-05

**Authors:** Jie Ning, Robert G. Beiko

**Affiliations:** Faculty of Computer Science, Dalhousie University, 6050 University Avenue, Halifax, Nova Scotia B3H 4R2 Canada

**Keywords:** Human microbiome, Support vector machine, Feature selection, Phylogenetic kernel, Functional and phylogenetic prediction

## Abstract

**Background:**

The microbiota from different body sites are dominated by different major groups of microbes, but the variations within a body site such as the mouth can be more subtle. Accurate predictive models can serve as useful tools for distinguishing sub-sites and understanding key organisms and their roles and can highlight deviations from expected distributions of microbes. Good classification depends on choosing the right combination of classifier, feature representation, and learning model. Machine-learning procedures have been used in the past for supervised classification, but increased attention to feature representation and selection may produce better models and predictions.

**Results:**

We focused our attention on the classification of nine oral sites and dental plaque in particular, using data collected from the Human Microbiome Project. A key focus of our representations was the use of phylogenetic information, both as the basis for custom kernels and as a way to represent sets of microbes to the classifier. We also used the PICRUSt software, which draws on phylogenetic relationships to predict molecular functions and to generate additional features for the classifier. Custom kernels based on the UniFrac measure of community dissimilarity did not improve performance. However, feature representation was vital to classification accuracy, with microbial clade and function representations providing useful information to the classifier; combining the two types of features did not yield increased prediction accuracy. Many of the best-performing clades and functions had clear associations with oral microflora.

**Conclusions:**

The classification of oral microbiota remains a challenging problem; our best accuracy on the plaque dataset was approximately 81 %. Perfect accuracy may be unattainable due to the close proximity of the sites and intra-individual variation. However, further exploration of the space of both classifiers and feature representations is likely to increase the accuracy of predictive models.

**Electronic supplementary material:**

The online version of this article (doi:10.1186/s40168-015-0114-5) contains supplementary material, which is available to authorized users.

## Background

Marker-gene profiles of human microbiota can provide a detailed view of microbial diversity across many body sites [1]. Body sites typically show very distinctive profiles; for example, healthy human gut samples are dominated by *Bacteroidetes* and *Firmicutes*, while skin samples tend to be much richer in *Actinobacteria* and other groups [2–4]. Clustering and ordination approaches such as principal coordinates analysis (PCoA) can illustrate the differences among different classes of body site [5, 6]. Similarly, many medical conditions are associated with dysbiosis, which is readily detectible through changes in the diversity or composition of human-associated microbes [4, 7–10]. Distinguishing samples within a site, such as on the skin (e.g., volar forearm, plantar, foot) or in the oral cavity (e.g., plaque, throat, saliva) is often more difficult [11–14]. Understanding these finer-grained degrees of variation is critical for building models of healthy microbiota. Models that conflate different sites, or fail to distinguish successional patterns, may not be as sensitive in the detection of, for example, the transition from a healthy to diseased state.

The similarity of sites can be understood in a metacommunity framework [15] as a combination of selective factors and proximity. From a selective point of view, similar environmental conditions such as site pH, oxygen availability, or adhesion potential may support the growth of taxonomically similar sets of bacteria [16, 17]. Geographic proximity can support mass-effect models where microbes from one site are transferred to another via migration processes. Examples of these processes can include skin-to-skin contact within or between individuals [18] and the transfer of microbes within the oral cavity due to direct contact and salivary mixing [17, 19]. The oral microbiome provides a particularly interesting test case for the classification of biodiversity, for several reasons. First, many ecologically distinct sites including different types of plaque, different surfaces, and saliva are found in close proximity [12, 20]. The oral habitat is highly variable with frequent inputs of nutrients, often followed by mechanical removal of the biofilm (e.g., via tooth brushing). The oral microbiome is also subject to well-characterized successional patterns [12] and frequently transitions to a diseased state [7, 21].

Unsupervised approaches such as ordination and clustering build associations from the most salient patterns of variation in a dataset; these primary patterns may or may not correlate with the features of interest [1]. By contrast, supervised classification approaches use knowledge of features to train models that can draw on any pattern of co-variation in the data [22, 23] and may perform better than unsupervised approaches when between-sub-site variation is small. Supervised approaches have previously been used to classify human microbiota [22, 24–26], using species or operational taxonomic units (OTUs) to distinguish different types of samples. However, microbiome data are typically high-dimensional, with potentially thousands of OTUs observed in each sample. Feature selection aims to identify a subset of all features that are most promising for classification, thereby eliminating uninformative features and decreasing the running time for the classifier [27]. Even when the accuracy of a classifier is not substantially improved, feature selection can still reveal key species or molecular functions of particular biological interest, because only the set of features that are most useful to classification (typically a very small subset of all features) is retained.

Supervised methods are effective for many classification problems; however, many previous studies took all oral samples as one class and tried to distinguish them from microbes from body sites such as skin or gut [22, 24]. One area of potential improvement is the augmentation of generic machine-learning techniques with biological and evolutionary insights. For example, support vector machines (SVMs) can base their classifications on customized similarity values between samples from the same or different body sites; distances such as UniFrac [28–30] can be informed by phylogenetic relationships among species or OTUs. Similarly, the use of OTUs in classification builds on an assumption that groups of closely related organisms can be treated as functional or ecologically cohesive units. This assumption may be violated by strain-level variation and conversely may apply to aggregations of clades that are broader than a single OTU, which again suggests a phylogenetic approach. Finally, the recently developed PICRUSt [31] algorithm can map taxonomic samples to functional profiles, based on known gene repertoires of closely related organisms: although less informative than shotgun metagenomic sequencing, such functional-prediction approaches may be more informative than taxonomic ones if key functions are decoupled from phylogenetic similarity due to processes such as lateral gene transfer and convergence. Transferred functions can become characteristic traits of phylogenetically distinct lineages [32] and PICRUSt can potentially identify sets of clades whose similarities are functional rather than phylogenetic. Some of these approaches yield significant increases in classification accuracy, while feature selection highlights key phylogenetic and functional features. We have implemented these ideas in a machine-learning framework and used oral microbiome samples from the Human Microbiome Project [33] as a challenging test case.

## Methods

### Dataset and sequence preprocessing

We obtained the oral microbiome marker-gene dataset from the Human Microbiome Project Data Analysis and Coordination Center (HMP DACC) [34] in February 2014. The oral dataset was originally collected from 242 volunteers ranging from 18 to 40 years old and included samples from nine sites within the oral cavity: saliva, supragingival and subgingival plaque (plaque above and below the gingival margin), tongue dorsum (top surface of the tongue), hard palate (roof of the mouth), buccal mucosa (inside lining of the cheek), attached keratinized gingiva (gums covering the jaw bones), and palatine tonsils (sides at the back of the throat) (see Additional file 1 drawn by SitePainter [35]). Sequences in this reference dataset included amplified regions of the V1–V3 and V3–V5 regions of the 16S rRNA gene (Additional file 2), although there were more sequences associated with the V3–V5 region (Table 1). In initial trials, we found that the V3–V5 region gave accuracy scores between 5 and 10 % higher than V1–V3, and focused on the better-performing region in the rest of this work.

**Table 1:** Details of human oral cavity samples from HMP, with associated abbreviations

Sub-sites	Acronym	Samples	OTUs	Seqs/sample (mean ± sd)	OTUs/sample (mean ± sd)
Saliva	SAL	281	6166	8596 ± 6034	521 ± 183
Attached keratinized gingiva	GING	304	3741	8998 ± 5756	313 ± 105
Buccal mucosa	BUCC	301	5370	9465 ± 10,268	447 ± 166
Hard palate	HPAL	300	5848	8935 ± 6575	441 ± 154
Palatine tonsils	PTON	304	5339	9586 ± 7247	448 ± 146
Throat	THRO	301	6278	9053 ± 7233	422 ± 147
Tongue dorsum	TONG	305	4400	10,351 ± 10,450	398 ± 129
Subgingival plaque	SUB	301	5782	9877 ± 5926	495 ± 147
Supragingival plaque	SUPRA	305	5277	10,413 ± 6564	497 ± 152

The pre-processed dataset with barcode and primer sequences removed was obtained directly from the HMP DACC (see Table 1 for summary statistics). All samples were processed using QIIME version 1.8.0 [36]. Sequences were clustered into OTUs at 97 % similarity through UCLUST version 1.2.22q [37], using a closed-reference OTU-picking strategy with GreenGenes (gg_13_08) as our reference database [38]. The resulting table contained the counts of each identified OTU in each sample. To account for disparities in OTU counts in different samples, we converted raw counts to proportions for each sample. Each of the nine oral cavity sites was used as the attribute label to be predicted, with relative OTU abundance as the potential predictors or features. The relative abundance was scaled such that the largest value in each sample was set to 1.0.

### Phylogenetic beta diversity

Sequences were aligned using QIIME’s default alignment method PyNAST version 1.2.2q [39], which implements the NAST alignment algorithm in Python. Sequences with alignment length <150 nucleotides or <75 % identity with the reference dataset were removed. A phylogenetic tree of OTUs was generated from the sequence alignment using FastTree version 2.1.3q [40]. Trees were visualized with ETE version 2.1 [41].

We used 14 non-phylogenetic and phylogenetic beta-diversity metrics to calculate the distance between each pair of samples with QIIME. To visualize the dissimilarity of the samples, PCoA was performed to visualize clusters of samples in a low-dimensional space. We also used the Unweighted Pair Group Method with Arithmetic Mean (UPGMA) approach to build hierarchical clusters that the group samples by their similarity.

#### Feature representation and selection

**Fig. 1:**
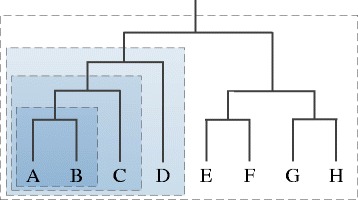
Generation of clade-based features. Each clade in the tree corresponds to a feature in the data set; for example, the darkest box encompasses OTUs A and B

OTUs can be useful entities for classifying microbial communities, but a strict OTU-based approach cannot directly use phylogenetically cohesive subsets of lineages within an OTU, or groups of OTUs, as predictors. To address this limitation, we developed a clade-based approach to feature generation (Fig. 1). In the original phylogenetic tree, which was rooted on the branch from *Archaea* to *Bacteria*, each of the leaves is a single OTU. Any set of two or more OTUs that constitutes a clade in the tree can also be represented as a feature, with the relative abundance of this group expressed as the sum of the frequencies of its constituent OTUs. The script to produce clade-abundance features can be found in Additional file 3. Since the number of non-leaf clades is equal to the number of internal nodes in the phylogenetic tree, this clade-based approach can generate a total of *l* − 2 features, where *l* is the number of leaves in the tree, if the uninformative root clade that includes all OTUs is ignored.

In addition to phylogenetic features such as taxon and clade abundance, we also generated functional predictions to use as input features. PICRUSt is a tool that can predict the functional repertoire of genomes associated with environmental sequences by mapping the content of closely related sequenced genomes. We used PICRUSt 1.0.0 to predict functions based on the previously constructed OTU tree to predict the functional trait abundance. The Kyoto Encyclopedia of Genes and Genomes (KEGG) orthology group descriptions [42] were used as the basis for functional predictions. As we did with OTUs, function counts were set to a maximum of 1.0 in each sample. We used the nearest sequenced taxon index (NSTI) of PICRUSt to estimate the reliability of functional predictions. NSTI sums all the branch lengths separating each OTU and its respective nearest sequenced genome, weighting for the relative abundance of each OTU in the community. Community members that have no close sequenced relatives will make a larger contribution to the NSTI score and have predictions that are in general less reliable.

Taxonomic and functional mapping generated thousands of features, many of which are likely to be uninformative, and in aggregate, can reduce the speed and accuracy of SVM training. Although some species appear in only a small number of samples, rare features may nonetheless be useful for classification and should not be removed by default. We used feature selection to accelerate learning by removing uninformative OTUs. Among the multitude of available feature selection techniques, we used two types of approach: *filter* methods, which consider the usefulness of features (often one at a time) based on their apparent relevance to the classification problem, and *wrapper* methods, which assess features by quantifying their effect on the accuracy of a trained model. The filter methods used were information gain, which ranks the features based on the amount of predictive information obtained from the presence or absence of a term [43] and the chi-square (*Χ*^2^) statistic, which quantifies the extent of correspondence between a feature and the class label [44]. Filter methods are fast and suitable for problems of high dimensionality but are independent from classifiers and often ignore interactions between features. We also considered random forest (RF) feature permutation [45] as a wrapper method. In this approach, variables were ranked based on the effect of randomizing their values between the categories to be predicted. In the context of a trained RF classifier, randomizing a useful variable would lead to a significant drop in accuracy, whereas a similar procedure on an uninformative variable would have no effect. Although OTUs with strong marginal effects (i.e., those that have good predictive power independent of any other variables) should be identified by all three approaches, useful combinations of variables might be highlighted by the RF approach.

### Classification using SVMs

SVMs have been widely used in various applications since their introduction by Cortes and Vapnik in 1995 [46]. SVMs are model-based classification methods that try to maximize the width of a decision boundary between categories. This decision boundary or hyperplane is typically defined by a small number of boundary cases (the *support vectors*) with relatively small distances to cases of the other type. A key attribute of SVMs is their ability to accept any similarity values that satisfy a set of constraints; the “kernel trick” allows mapping of cases into a higher-dimensional space where the linear SVM classifier can perform well.

Classification was performed using the LIBSVM package [47]. We chose the generic one-parameter radial basis function (RBF) kernel for classification. To pick the best combination of kernel width *γ* and error penalty parameter *C*, a grid search using every combination of *C* and *γ* was done (finite sets of attempt values for *C* = [log_2_^−5^, log_2_^15^], *γ* = [log_2_^−15^, log_2_^3^]). A fivefold cross-validation approach was adopted to evaluate the classification models. This cross-validation procedure was repeated 100 times for each trial, each time using a different random number seed, in order to generate distributions of accuracy scores.

Generic polynomial and radial basis function kernels are widely used, but custom kernels that incorporate biological insights can be useful as well. For example, alignment-based kernels improved SVM performance in subcellular protein localization prediction [48, 49]. Since phylogenetic distance is an effective measure in the comparison of microbial communities, we developed a custom kernel based on the weighted and unweighted UniFrac distances. We also constructed several non-phylogenetic beta-diversity kernels including Bray-Curtis and Euclidean distance (Additional file 4). Since beta-diversity expresses the dissimilarity between each pair of samples, we subtracted each such value from 1.0 in order to generate similarity values for the SVM classifier. These similarity scores were combined with several different OTU table preprocessing approaches, including raw OTU count, relative abundance, rarified counts from 500 to 3000 per samples, and cumulative sum scaling (CSS) normalization [50].

Although our focus was on SVMs, we also considered two other supervised classification methods, SourceTracker [23] and RF. SourceTracker is a Bayesian approach that assigns probabilities that a given sample is derived from each of a set of environment types. By calculating the posterior probability of each source environment assignment with Gibbs sampling, SourceTracker gives probabilities of where a sink sample came from. We used SourceTracker version 0.9.5 software as implemented in QIIME with default settings. Analogous to fivefold cross-validation, the set of samples was divided into five subsets: one subset was used as sink samples while the other four are source samples. We repeated this process five times with different cross-validation subsampling. RFs, first introduced in 2001 [51], are an ensemble method merging decision trees with voting schemes. Each decision tree is constructed based on M (mtry) randomly chosen features from the input dataset. The prediction of every sample is determined by the majority vote of all these decision trees. RF classifiers are popular both for feature selection and classification and were found by Knights et al. [22] to perform well on several test datasets. RF classification was implemented with scikit-learn 0.15 [52].

## Results and discussion

### OTU diversity across nine oral cavity sites

A total of 2706 human oral cavity samples from nine oral sites were collected from the HMP database. The samples covered the V3–V5 region of the 16S rRNA gene (Table 1, Additional file 2). All sites had at least 281 associated samples. The total OTU richness across all samples of a given site varied from a minimum of 3741 (attached keratinized gingiva) to over 6000 (saliva and throat). The average number of sequences per sample ranged from approximately 8500 to 11,500, although the variation within each site was high.

### Classification of all oral cavity sites

**Fig. 2:**
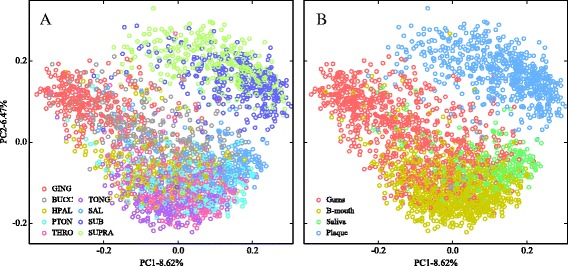
Principal coordinates analysis of nine oral cavity sites. The same data set is shown with all nine oral cavity sites (**a**) and four clustered groups (**b**) as labels. Distances were computed using the unweighted UniFrac distance

We generated PCoA plots based on unweighted UniFrac distances between samples to visualize the separation of points between the nine sample types (Fig. 2a). The first two principal coordinates explain 15.07 % of the total variance in the data set and do not provide clear separation of any of the nine sample types. Clustering patterns are nonetheless visible in the figure; in particular, the supragingival and subgingival plaque samples constitute a group that is largely separate from the other sample types. The other seven sample types occupy one large cluster, but none of these is uniformly distributed throughout the cluster: for example, the attached keratinized gingival samples tend to have negative values in principal coordinate one and near-zero values in principal coordinate two.

**Fig. 3:**
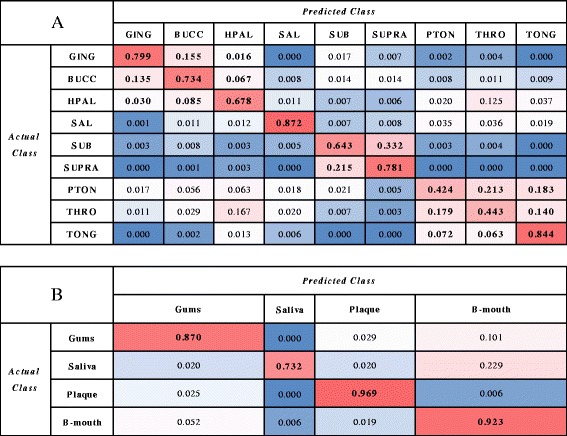
Confusion matrix of nine-way oral site classification. *Rows* indicate the correct label for each sample, while *columns* indicate the label predicted by the classifier. Each cell indicates the number of samples of a given type classified to each sample type. The classification patterns of all nine classes (**a**) and a recoding into four classes (**b**) are shown

We performed SVM classification using an RBF kernel on all 2702 oral cavity samples. The cross-validated classification accuracy with respect to the sample type label was 69.73 %. The confusion matrix (Fig. 3a), which shows the frequency with which samples of a given type were correctly classified or misclassified to another category, shows a non-random pattern of misclassification. Of the nine oral cavity sites, saliva and tongue dorsum were classified with the highest accuracy (87.2 and 84.4 %, respectively), while samples from the palatine tonsils and throat were correctly classified less than 45 % of the time. We identified four natural groupings of the sites based on these patterns. Ninety percent of the samples from the attached keratinized gingiva, buccal mucosa, and hard palate group were most often classified within the same group, which we define as “back of the mouth”; the misclassification of 12.5 % of hard palate samples to the throat represents the only major confusion between this group and any other. Consistent with the separation seen in Fig. 2a, 98.6 % of the subgingival and supragingival plaque samples are classified as one of these two sites. The samples from the throat, palatine tonsils, and tongue dorsum constitute another group responsible for 85.4 % of all classifications, although the throat and tonsils are also conflated with the hard palate and buccal mucosa. Finally, the salivary samples are not misclassified as any other site at a rate higher than 3.6 %. In general, these four groups consist of sites that are proximal in the mouth, corresponding roughly to gums (attached keratinized gingiva, buccal mucosa, and hard palate), plaque (supragingival and subgingival plaque), back of the mouth (throat, palatine tonsils, and tongue dorsum), and saliva. Because of the gag reflex, collecting samples from throat is the most difficult work among the nine sites. Samples are easy to be contaminated during the depressor getting back from throat, so throat samples may be mixed with hard palate microbes [53].

Recoloring sample points in the original PCoA plot to reflect the four groups (Fig. 2b) shows the clearer distinction among sites, albeit still with a substantial amount of overlap among all but the plaque group. The nine sites were recoded into their four constituent categories and once again classified using an SVM with the RBF kernel (Fig. 3b). The classification accuracy of plaque samples is 96.9 %, as compared with 73.2 % accuracy for saliva, 87.0 % for gums, and 92.3 % for the back of the mouth. Since the plaque samples were well separated from the other groups, but difficult to distinguish based on the confusion matrix in Fig. 3a, we chose to focus on this two-class problem in order to try and improve the classification accuracy for a challenging subset of sites.

### Distinguishing plaque samples using taxonomic information

Studies culturing the microbial communities in hard plaque detected taxonomic differences between these two sites [12]; one key observation was that aerobic bacteria were more abundant in the supragingival than the subgingival plaque [54]. PICRUSt demonstrated similar results by predicting more metabolic citrate cycle genes in the supragingival plaque [31]. However, these reported trends do not completely distinguish the two sites, and although plaque samples are clearly isolated from other sites in the PCoA plot, the similar flora within plaque makes it difficult to distinguish the supragingival from the subgingival samples [55, 56]. A binary classification of the subgingival and supragingival samples without any feature selection was performed using an SVM trained with an RBF kernel. A total of 7048 OTUs was included in the model (Additional file 5), which achieved a cross-validated accuracy of 76.2 % (Table 2). All three feature-selection methods yielded higher classification accuracy, with information gain and chi-square yielding maximum accuracy scores of 77.9 % with 60 features and 77.7 % with 50 features, respectively, and RF feature permutation showing the best performance with an accuracy of 79.8 % on only 20 features. It appears that the feature permutation-based wrapper approach is able to identify features that are complementary, while a straight ranking of features using the filter approaches miss many of these complementary features that may have lower marginal performance. The performance of RF feature permutation is highest with 10–20 features, and performance drops off to a level more consistent with the filter methods as more features are added.

**Table 2:** Accuracy of SVM classifiers trained with different combinations of input features

	Cross-validation accuracy (number of features)			
Type of input features	Without feature selection	With feature selection		
		Info_Gain	Chi-square	Feat_Perm
(i) OTU	0.762 (7048)	0.779 (60)	0.777 (50)	0.798 (20)
(ii) Clade	0.738 (14,402)	0.802 (110)	0.800 (170)	0.802 (100)
(iii) Function	0.761 (6191)	0.762 (120)	0.754 (100)	0.761 (60)
(iv) Hybrid	0.777 (1556/1518)	0.804 (92/78)	0.805 (68/62)	0.805 (28/22)

The initial numbers show the accuracy score, with numbers in parentheses indicating the total number of features used to train and test the classifier. The four types of input features used were (i) OTUs only, (ii) OTUs and clades comprising related sets of OTUs, (iii) functional predictions made using PICRUSt, and (iv) a dataset comprising all generated features. Feature selection techniques used were the filter methods, information gain and chi-square, and the feature permutation wrapper method

### SVM with custom beta-diversity kernels

We developed custom kernels based on 14 different beta-diversity measures that express the similarity between all pairs of samples. The hypothesis underlying the use of these kernels is that similarity scores based on ecological similarity measures will outperform a naïve RBF kernel, especially when these measures are based on information not available to the classifier (for example, phylogenetic information in the case of UniFrac). The performance of SVMs with different custom kernels and OTU table preprocessing approaches is given in Additional file 4. Colors are consistent with [5], which identified subsets of beta-diversity measures that gave highly correlated predictions. Phylogenetic measures did not yield improved accuracy relative to non-phylogenetic measures: for example, the widely used unweighted and weighted UniFrac measures yielded 74.4 and 73.7 % accuracy. The Canberra distance, recommended by Kuczinski et al. [57], yielded an accuracy score of 76.5 %, an improvement on UniFrac but still worse than using OTU abundance with an RBF kernel. For the OTU tables used in calculating distance, we pre-processed them with four different methods. CSS normalization was able to separate different samples well, especially for Euclidean distance. OTU table rarefaction produced the lowest score and largest deviation. Although many types of microbial samples cluster well based on beta-diversity measures such as Bray-Curtis or UniFrac, this is clearly not the case with the two types of plaque. A possible reason for the discrepancy between RBF and our custom kernels is the optimization of the gamma parameter of the radial basis function in the SVM grid search, whereas none of the 14 beta-diversity measures have a free parameter that can similarly be optimized.

### Classification with clade-abundance features

**Fig. 4:**
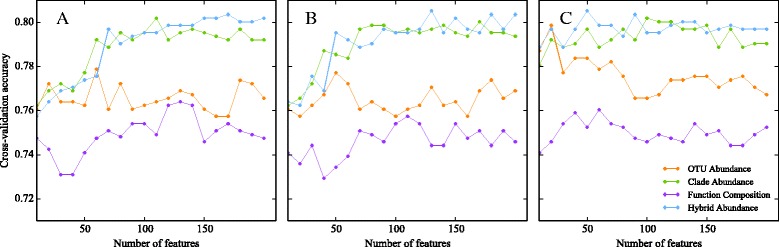
Classification accuracy with different sets of input features. The classification accuracy is shown for sets of 10 to 200 of the top-ranked features according to the information gain (**a**), chi-square (**b**), and RF feature permutation (**c**) criteria. The four types of input features used were OTUs only (*orange markers*), OTUs and clades (*green markers*), functional predictions made using PICRUSt (*purple markers*), and all generated features (*blue markers*)

Since the beta-diversity custom kernels did not improve the classification accuracy, we used the generic RBF kernel in all subsequent trials. We next augmented the OTU table with relative abundance information about clades that contain multiple OTUs, to determine whether explicit specification of relationships among OTUs might lead to better prediction accuracy. Fifty-two OTUs were lost because their corresponding sequences failed the PyNast quality control filters, leaving a total of 6996 OTUs. To this set, we added 6994 clades, corresponding to all internal nodes in the reference tree, minus the uninformative root node which always has a relative abundance of 1.0. The classification accuracy obtained without feature selection was less than that obtained from the OTU table without clade information (73.8 vs. 76.2 %). While the OTU + clade table has almost twice as many features than the OTU abundance table alone and includes over 99 % of the original OTUs (Additional file 6), it appears that the higher dimensionality of the data confounds the SVM classifier, making it more difficult to build an accurate model. However, applying feature selection as above gave at least 80 % accuracy (Table 2). As was observed previously with the OTU table, the filter methods required more features to achieve their maximum classification accuracy (110 and 170 for information gain and chi-square versus 100 features for RF approach). Figure 4 shows the performance of classifiers with different numbers of features. Information gain (Fig. 4a) and chi-square (Fig. 4b) had similar performance: the accuracy of OTU abundance varied between 76 and 78 % with different numbers of features. However, clade abundance gave accuracy scores that were often in excess of 80 %. Both OTU and clade abundance can classify samples well with a small number of RF-ranked features (Fig. 4c), but with the number of features increasing, the performance of OTU abundance worsened whereas clade abundance kept working well. It appears that explicitly modeling the phylogenetic correlations between OTUs allows the filter methods to exploit the interactions that were previously accessible only to the wrapper method.

**Fig. 5:**
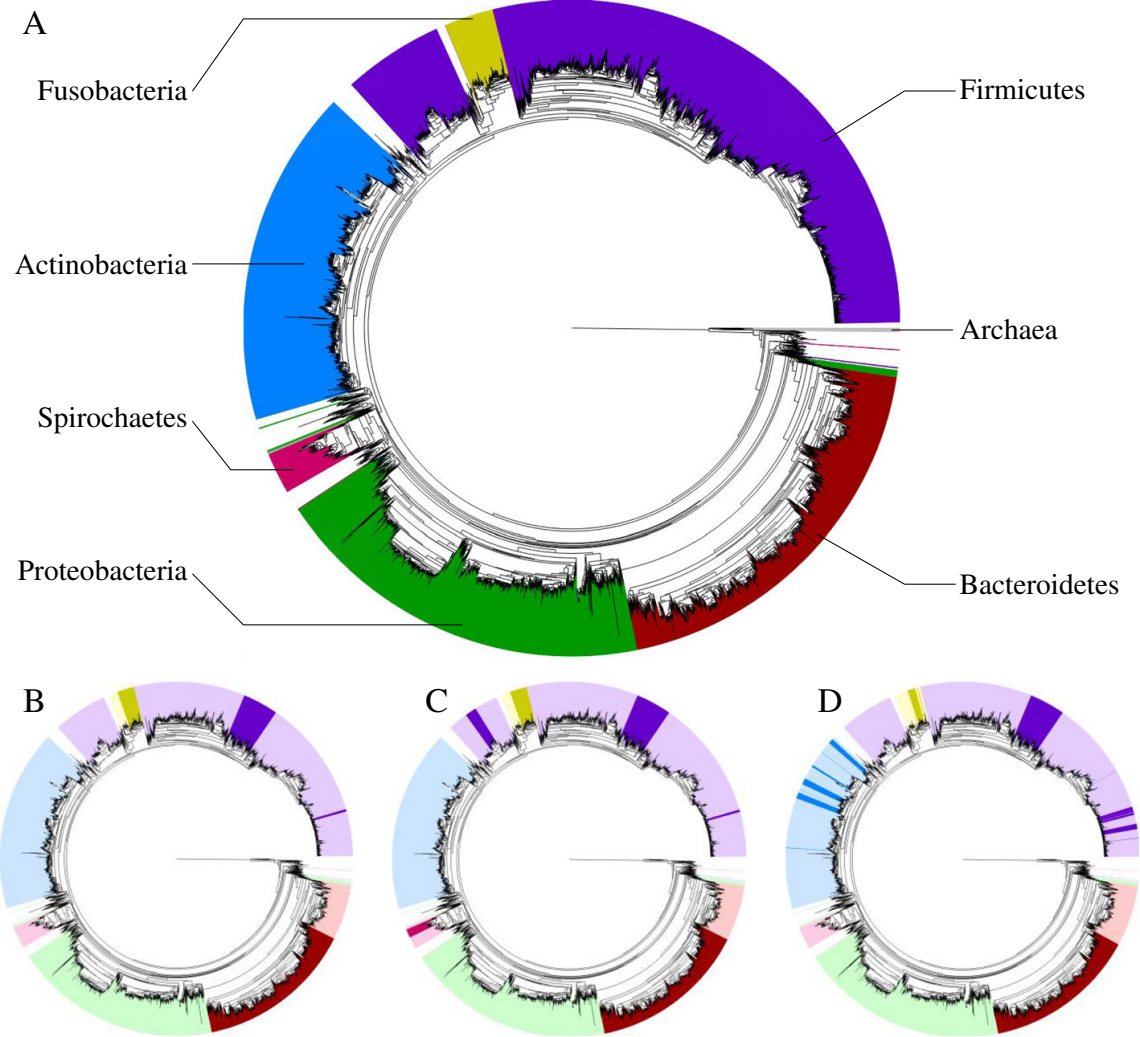
Phylogenetic mapping of top-ranked clade and OTU features. **a** Reference tree comprising all observed oral site OTUs, with branch lengths proportional to substitutions per site. Key phyla are highlighted with *different colors*. **b**–**d** Mapping of the highest-ranked clade and OTU features according to information gain (**b**, 110 features), chi-square (**c**, 170 features), and RF feature permutation (**d**, 100 features)

The phylogenetic mappings and corresponding GreenGenes taxonomic classifications of OTUs are shown in Fig. 5a (Additional file 7). The subgingival plaque samples tended to have higher proportions of *Bacteroidetes* (sub: 0.254 vs supra: 0.191), *Fusobacteria* (0.172 vs 0.118), and *Spirochaetes* (0.029 vs 0.006), whereas *Actinobacteria* (0.175 vs 0.247) and *Proteobacteria* (0.151 vs 0.215) are more abundant in the supragingival plaque. *Firmicutes* had similar abundance in both types of site (0.213 vs 0.220); however, at the class level, *Bacilli* (0.110 vs 0.148) and *Clostridia* (0.103 vs 0.071) showed larger deviations.

We then highlighted the optimal features that were selected by each method in the phylogenetic tree. The filter methods, information gain (Fig. 5b), and chi-square (Fig. 5c) chose similar clades including a large clade within *Bacteroidetes* and smaller groupings within *Firmicutes* and *Fusobacteria*. The chi-square approach chose the largest number of features, including *Spirochaetes* and *Clostridia* clades that were not chosen by the information gain criterion. By contrast, the RF feature permutation approach, which included the fewest features in its optimal set, selected a greater diversity of features (Fig. 5d). This set of features included unique clades of *Firmicutes* and *Actinobacteria* that were not identified by the information gain or chi-square approaches. For all three feature selection methods, near-optimal classification accuracy was obtained for many different numbers of selected features, suggesting that some of the highlighted clades in Fig. 5 may not be important for classification purposes. Nonetheless, the higher variety of features selected by the RF feature permutation approach shows the value of testing combinations of features during the selection process.

### Classification based on predicted functional profiles

The PICRUSt software allows the prediction of functional gene complements in microbial samples that have been characterized with marker genes such as 16S. We used these predictions as the basis for classification: if the functional capacity of microbes in a system is more important than their specific taxonomic affiliations, then a function-based approach to classification may yield higher accuracy. PICRUSt uses phylogenetic information to make its predictions, and thus functional information will be highly correlated with the OTU and clade data. However, since phylogenetically distant lineages can share common functional features, the predictions made by PICRUSt may identify functional similarities between OTUs that belong to different high-level taxonomic groups such as classes and phyla. Thus, the predictions made by PICRUSt are not completely redundant with the OTU and clade features considered in this work.

To measure the reliability of the functional predictions, we calculated the NSTI values for each sample (mean NSTI = 0.04 ± 0.01 sd). This is similar to the values reported for HMP samples (mean NSTI = 0.03 ± 0.02 sd), which were generally well predicted by PICRUSt, as compared with 0.23 ± 0.07 for a less well-predicted hypersaline community. A total of 6191 KEGG orthologs, which incorporate functional predictions in addition to homology information, were used as input features to an SVM with an RBF kernel as performed above (Additional file 8). The cross-validated accuracy of the model trained with all features was 76.1 %, very similar to the value obtained with the corresponding OTU abundance model. These observations are consistent with those of Xu et al. [58], who found that taxonomy alone was sufficient to model microbial community structure. Functional features are still useful for predictive purposes, but their failure to improve classification accuracy may be attributable to several factors. It may be that the crucial functions are not well annotated by KEGG, because of misannotations or a failure to assign to any meaningful functional category. The granularity of KEGG functional attributions and the presence of irrelevant features may also impede the discovery of important predictive attributes.

**Fig. 6:**
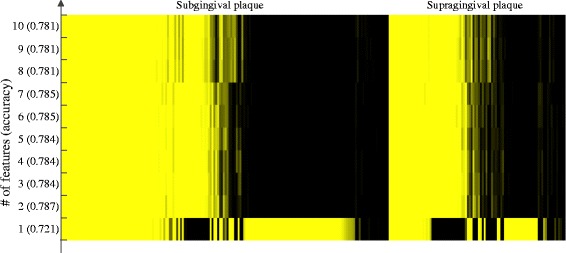
Classification of plaque samples using the 1–10 top-ranked features. The heatmap of times that samples were misclassified during 100 replicated runs. The rows correspond to the number of features used and the corresponding accuracy. Each column represents one sample, a *yellow bar* indicates a sample was misclassified in all 100 replicates, while a *black bar* indicates that a sample was correctly predicted in all replicates. Only those samples that were misclassified at least once are shown

Since both function and taxonomy yield similar classification accuracy, combinations of the two types of feature may further improve accuracy if they contain complementary information. To assess the performance of classifiers based on combined clade and functional information, we performed feature selection on a hybrid data set containing features of both types. The results of feature selection and classification are shown in Table 2. The accuracy obtained from all three types of feature selection was 80.4–80.5 %, and the RF feature permutation approach yielded a maximum accuracy score with only 28 clade-based and 22 functional features. The small improvement in accuracy of the hybrid approach relative to clade-based classification alone (Table 2, Fig. 4) suggests that the functional features do not provide much useful complementary information to taxonomy: the increase of 0.3 % relative to previous wrapper-based results corresponds to only a few additional correctly classified cases. However, the small number of selected clade-based and functional features that yield these accuracy scores allow us to focus on the key attributes that distinguish the two types of plaque. To focus on a smaller set of features, we defined groups of features with high correlations (Spearman *r* ≥ 0.5) and chose only the highest-ranking or *primary* feature from each group, thus preserving relevance while reducing feature redundancy. SVMs built from the top ten features showed slightly reduced accuracy in comparison with the overall performance of clade and hybrid features but demonstrated the central importance of a very small number of features (Fig. 6). Intriguingly, a number of samples were correctly classified when the single best feature (a clade of *Streptococcus*) was used but incorrectly classified when one or more additional features were included.

Three of the top ten groups of features included only functional features (KOs), while the other seven included at least one taxonomic and one functional feature (Additional file 9). Two groups, including the group ranked first in feature selection, were restricted to *Streptococcus*, with several clades in this group restricted to the opportunistic pathogen *Streptococcus anginosus*. Two additional groups included other clades of *Streptococcus*, underscoring the importance of different members of this genus in the oral cavity. Although *Streptococcus* is typically a more significant component of supragingival plaque, consistent with its facultative anaerobic lifestyle, three of the *Streptococcus*-containing groups were overrepresented in subgingival plaque, while the fourth was 50 % more abundant in supragingival plaque. This finding suggests that the most common types of *Streptococcus* may not be the best discriminators between the two types of plaque. Other selected groups of features were broader in their taxonomic distributions, although some of these groups included genera such as *Prevotella*, *Fusobacterium*, and *Dialister*. The second-ranked group included eight genera (including *Streptococcus*) as well as a number of higher clades, which suggests a set of co-occurring and possibly interacting bacteria that are characteristic of subgingival plaque.

The best feature group included a single functional class, *sagA*, which encodes the basic structural unit of *Streptolysin S* (SLS). Bacteria such as *Streptococcus pyogenes* use SLS to lyse host cells and acquire iron [59, 60]. By its strong correlation with the primary feature in group 1 (Spearman *r* = 0.97, *p* < 10^−250^), this function appears to be strongly associated with subgingival plaque. Functions that were either the primary feature for their group or had the best correlation with the primary feature of their group included a beta-lactam resistance protein, overrepresented in subgingival plaque: streptokinase, which can aid the spread of *Streptococcus* infection through cleavage of fibrils [61]; proteins involved in resistance to tellurium and vancomycin; and a type IV secretion system component. Although many of the implicated functions relate to host-microbial interactions, we found no clear, strong connections to aerobic or anaerobic lifestyles.

### Comparison with other supervised learning methods

RFs were also implemented with sets of 10 to 200 features ranked highly by the three feature selection methods. The running time of SourceTracker was much higher, and we considered only the entire set of features when testing its performance. For the RF model, the lowest and highest accuracies were respectively achieved by functional abundance and hybridization features, consistent with the SVM results (Additional file 10). OTU and clade representations had similar performance when features were ranked by information gain (Additional file 10A) and chi-square (Additional file 10B), but clade abundance had improved accuracy when RF feature permutation (Additional file 10C) was used to rank features. SourceTracker is able to estimate the proportion of possible sources for each sample; the source with the largest associated probability was used as the final prediction. SourceTracker models trained on OTU features outperformed all other representations, suggesting that redundant features impeded the accuracy of the classifier.

Both SourceTracker and RF had a similar performance on the plaque datasets, with a classification accuracy between 75 and 78 % when OTU abundance features were used. However, the three methods correctly classified overlapping but non-identical subsets of all plaque samples. Additional file 11 compares the classification of all samples for each pairwise combination of methods. The plots show all three methods had consistent predictions for most of the samples, but a small subset of samples were consistently correctly predicted by one approach and incorrectly predicted by the other. This pattern suggests that “ensemble” methods which combine the predictions of multiple classifiers may be able to outperform individual classifiers.

## Conclusions

A primary objective of machine learning is to train models that can distinguish classes of entities, in this case, microbial samples encoded as OTU tables, with a high accuracy. In our examination of oral cavity samples, the best test set accuracy scores we have obtained are on the order of 80–81 %, which demonstrates useful learning but is of little value for diagnostic applications and is probably not suitable as a reference model for comparison with diseased states, for example, unless it is satisfactory to use both plaque types as a single reference group. Previous authors have tested many different machine-learning algorithms on reference data sets [22, 24–26, 58]; our exclusive focus here on SVMs allowed us to consider different encodings of the input data and biologically inspired kernels in detail. Of the modifications we tried, clade-based representations gave the largest increase in performance. Although the combinations of OTUs that constituted clades could in principle be discovered by the classifier, it is clear that explicit clade representations yielded some advantage in both feature selection and classification. Selected clades contained genera known to be important in the human oral cavity, in particular *Streptococcus*. Our predictive approach to function did not improve the accuracy of our classifiers, in spite of the potential for PICRUSt to identify functional as well as phylogenetic connections between OTUs and clades. It may be that shotgun metagenome sequencing, which generates accurate information about even those genes that are frequently transferred, may yield a higher predictive accuracy.

Why did we not obtain higher accuracy scores? Previous work suggests that a different choice of classifier or data encoding may yield higher classification accuracy; clearly, further work is needed to explore this question, and there is a multitude of different approaches that can be applied to the data. Changing the definition and inference of OTUs may improve performance as well: in particular, changing the OTU threshold from 97 to 99 % would highlight finer-scale differences in abundance, for example, differences that may manifest only at or below the species level. In this work, we used closed-reference OTU picking because it maps sampled sequences to reference groups that are defined prior to the analysis. However, closed-reference picking discards any sequences that do not map to existing OTUs at the required level of sequence similarity, a phenomenon that is especially acute at higher thresholds such as 99 %. An approach that combines closed-reference and de novo OTU generation would likely be ideal but requires that new OTUs be comparable between samples and across studies. An alternative would be to use methods that modify the OTU definition or dispense with it altogether such as SWARM [62] or approaches that compare observed sequences across multiple samples [63].

In the case of oral samples, and gingival samples in particular, complete separation (i.e., 100 % classification accuracy) may not be achievable, for several reasons. Chief among these is the physical proximity of the supragingival and subgingival plaque. Although the two sites are different in terms of nutrient and oxygen availability, among other factors, some amount of mixing is inevitable due to mass effects, even if microbes from one site are not viable in the other. Sample misidentification may also contribute to diminished classification; indeed, this was one identified use of SourceTracker. However, we expect the impact of misidentified samples to be very low, for two reasons: first, the HMP followed very strict protocols regarding the collection and handling of samples; second, the overlapping of sample types we see in Fig. 2 suggests a gradient of diversity from one sample type to the others, rather than a few scattered outliers that might be indicative of misclassified samples. It is also unlikely that there is a single type of “healthy” subgingival and supragingival microbial community, which would impede the ability of a classifier to learn a single, general model of classification.

## Additional files

Additional file 1:
**Human oral cavity diagram drawn by SitePainter [** 35 **].** Different oral sites are identified using different colors, and abbreviations used in the text are given. (PDF 9 kb) Additional file 2:
**Summary of microbiome samples.** Human oral cavity samples came from HMP with associated abbreviations. OTUs were picked using a closed-reference strategy with a 97 % identity threshold. (XLSX 32 kb) Additional file 3:
**Script to produce clade abundance features.** (ZIP 788 kb) Additional file 4:
**The performance of SVMs with different custom kernels.** The distance metrics are ranked by their mean values and highlighted with colors consistent to the similarity-based clustering of [5]. Highly correlated measures are grouped in one color set. (PDF 7 kb) Additional file 5:
**OTU counts raw dataset.** (TXT 8,494 kb) Additional file 6:
**Clade counts raw dataset.** (TXT 9,214 kb) Additional file 7:
**Taxonomic composition of samples.** The relative abundance of each group in subgingival and supragingival plaque is shown. Taxonomy is displayed from kingdom to species level. (XLSX 492 kb) Additional file 8:
**Functional raw dataset.** (TXT 12,193 kb) Additional file 9:
**Top ten group features.** Taxonomic and functional features with Spearman correlation > 0.5 are grouped together. (XLSX 48 kb) Additional file 10:
**Classification accuracy of RF and SourceTracker using different sets of input features.** On the left side of each plot is the RF classification accuracy with sets of 10 to 200 of the top-ranked features according to information gain (A), chi-square (B), and RF feature permutation (C) criteria. The right-hand side of each plot shows Source Tracker’s classification accuracy with all features. The four types of input features used were OTUs only (orange markers); OTUs and clades (green markers); functional predictions made using PICRUSt (purple markers); and all generated features (blue markers). (PDF 28 kb) Additional file 11:
**The predictions on all samples: (a) SVM vs random forests, (b) SVM vs SourceTracker.** The values on *x-* and *y*-axis indicate the frequency of samples that were correctly predicted by each of the two methods. The size of the nodes reflects the number of samples that were classified with the indicated accuracy, from 0 % by both classifiers in the lower left-hand corner to 100 % in the upper right-hand corner. (PDF 6,199kb)

## References

[1] Costello EK, Lauber CL, Hamady M, Fierer N, Gordon JI, Knight R (2009). Bacterial community variation in human body habitats across space and time. Science.

[2] Zhou Y, Gao H, Mihindukulasuriya KA, La Rosa PS, Wylie KM, Vishnivetskaya T (2013). Biogeography of the ecosystems of the healthy human body. Genome Biol.

[3] Schloss PD (2014). Microbiology: an integrated view of the skin microbiome. Nature.

[4] Cho I, Blaser MJ (2012). The human microbiome: at the interface of health and disease. Nat Rev Genet.

[5] Parks DH, Beiko RG (2013). Measures of phylogenetic differentiation provide robust and complementary insights into microbial communities. ISME J.

[6] Huse SM, Ye Y, Zhou Y, Fodor AA (2012). A core human microbiome as viewed through 16S rRNA sequence clusters. PLoS One.

[7] Galimanas V, Hall MW, Singh N, Lynch MDJ, Goldberg M, Tenenbaum H (2014). Bacterial community composition of chronic periodontitis and novel oral sampling sites for detecting disease indicators. Microbiome.

[8] Turnbaugh PJ, Hamady M, Yatsunenko T, Cantarel BL, Duncan A, Ley RE (2009). A core gut microbiome in obese and lean twins. Nature.

[9] Schmidt BL, Kuczynski J, Bhattacharya A, Huey B, Corby PM, Queiroz ELS (2014). Changes in abundance of oral microbiota associated with oral cancer. PLoS One.

[10] Wade WG (2013). The oral microbiome in health and disease. Pharmacol Res.

[11] Grice EA, Kong HH, Conlan S, Deming CB, Davis J, Young AC (2009). Topographical and temporal diversity of the human skin. Science (80-).

[12] Segata N, Haake SK, Mannon P, Lemon KP, Waldron L, Gevers D (2012). Composition of the adult digestive tract bacterial microbiome based on seven mouth surfaces, tonsils, throat and stool samples. Genome Biol.

[13] Ximénez-Fyvie LA, Haffajee AD, Socransky SS (2000). Comparison of the microbiota of supra- and subgingival plaque in health and periodontitis. J Clin Periodontol.

[14] Bik EM, Long CD, Armitage GC, Loomer P, Emerson J, Mongodin EF (2010). Bacterial diversity in the oral cavity of 10 healthy individuals. ISME J.

[15] Costello EK, Stagaman K, Dethlefsen L, Bohannan BJM, Relman DA (2012). The application of ecological theory. Science.

[16] Ding T, Schloss PD (2014). Dynamics and associations of microbial community types across the human body. Nature.

[17] Simón-Soro A, Tomás L, Cabrera-Rubio R, Catalan MD, Nyvad B, Mira A (2013). Microbial geography of the oral cavity. J Dent Res.

[18] Meadow JF, Bateman AC, Herkert KM, O’Connor TK, Green JL (2013). Significant changes in the skin microbiome mediated by the sport of roller derby. PeerJ.

[19] Kort R, Caspers M, Van De GA, Van EW, Keijser B, Roeselers G (2014). Shaping the oral microbiota through intimate kissing. Microbiome.

[20] Faust K, Sathirapongsasuti JF, Izard J, Segata N, Gevers D, Raes J (2012). Microbial co-occurrence relationships in the human microbiome. PLoS Comput Biol.

[21] Claridge JE, Attorri S, Musher DM, Hebert J, Dunbar S (2001). Streptococcus intermedius, Streptococcus constellatus, and Streptococcus anginosus (“Streptococcus milleri group”) are of different clinical importance and are not equally associated with abscess. Clin Infect Dis.

[22] Knights D, Costello EK, Knight R (2011). Supervised classification of human microbiota. FEMS Microbiol Rev.

[23] Knights D, Kuczynski J, Charlson ES, Zaneveld J, Mozer MC, Collman RG (2011). Bayesian community-wide culture-independent microbial source tracking. Nat Methods.

[24] Statnikov A, Henaff M, Narendra V, Konganti K, Li Z, Yang L (2013). A comprehensive evaluation of multicategory classification methods for microbiomic data. Microbiome.

[25] Wang Y, Zhou Y, Li Y, Ling Z, Zhu Y, Guo X (2012). An improved dimensionality reduction method for meta-transcriptome indexing based diseases classification. BMC Syst Biol.

[26] Liu Z, Hsiao W, Cantarel BL, Drábek EF, Fraser-Liggett C (2011). Sparse distance-based learning for simultaneous multiclass classification and feature selection of metagenomic data. Bioinformatics.

[27] Saeys Y, Inza I, Larrañaga P (2007). A review of feature selection techniques in bioinformatics. Bioinformatics.

[28] Lozupone C, Knight R (2005). UniFrac: a new phylogenetic method for comparing microbial communities UniFrac: a new phylogenetic method for comparing microbial communities. Appl Environ Microbiol.

[29] Lozupone C, Lladser ME, Knights D, Stombaugh J, Knight R (2011). UniFrac: an effective distance metric for microbial community comparison. ISME J.

[30] Chang Q, Luan Y, Sun F (2011). Variance adjusted weighted UniFrac: a powerful beta diversity measure for comparing communities based on phylogeny. BMC Bioinformatics.

[31] Langille MGI, Zaneveld J, Caporaso JG, McDonald D, Knights D, Reyes JA (2013). Predictive functional profiling of microbial communities using 16S rRNA marker gene sequences. Nat Biotechnol.

[32] Andam CP, Gogarten JP (2011). Biased gene transfer and its implications for the concept of lineage. Biol Direct.

[33] The NIH HMP Working Group (2009). The NIH human microbiome project. Genome Res.

[34] Human microbiome project [ftp://public-ftp.hmpdacc.org] Access February 4, 2014.

[35] Gonzalez A, Stombaugh J, Lauber CL, Fierer N, Knight R (2012). SitePainter: a tool for exploring biogeographical patterns. Bioinformatics.

[36] Caporaso JG, Kuczynski J, Stombaugh J, Bittinger K, Bushman FD, Costello EK (2010). QIIME allows analysis of high-throughput community sequencing data. Nat Methods.

[37] Edgar RC (2010). Search and clustering orders of magnitude faster than BLAST. Bioinformatics.

[38] DeSantis TZ, Hugenholtz P, Larsen N, Rojas M, Brodie EL, Keller K (2006). Greengenes, a chimera-checked 16S rRNA gene database and workbench compatible with ARB. Appl Environ Microbiol.

[39] Caporaso JG, Bittinger K, Bushman FD, Desantis TZ, Andersen GL, Knight R (2010). PyNAST: a flexible tool for aligning sequences to a template alignment. Bioinformatics.

[40] Price MN, Dehal PS, Arkin AP (2009). Fasttree: computing large minimum evolution trees with profiles instead of a distance matrix. Mol Biol Evol.

[41] Huerta-Cepas J, Dopazo J, Gabaldón T (2010). ETE: a python environment for tree exploration. BMC Bioinformatics.

[42] Kanehisa M, Goto S, Sato Y, Furumichi M, Tanabe M (2012). KEGG for integration and interpretation of large-scale molecular data sets. Nucleic Acids Res.

[43] Yang Y, Pedersen JO (1997). A comparative study on feature selection in text categorization. Mach Learn Work Then Conf.

[44] Zheng Z, Wu X, Srihari R, Srihani R (2004). Feature selection for text categorization on imbalanced data. ACM SIGKDD Explor Newsl.

[45] Altmann A, Toloşi L, Sander O, Lengauer T (2010). Permutation importance: a corrected feature importance measure. Bioinformatics.

[46] Cortes C, Vapnik V (1995). Support-Vector Networks. Mach Learn..

[47] Chang C-C, Lin C-J (2011). LIBSVM. ACM Trans Intell Syst Technol.

[48] Davis L, Hawkins J, Maetschke SR, Boden M (2006). Comparing SVM sequence kernels: a subcellular localization theme. 2006 Work Intell Syst Bioinforma (WISB 2006).

[49] Chen J, Li H (2013). Topics in applied statistics. Springer Proceedings in Mathematics & Statistics.

[50] Paulson JN, Stine OC, Bravo HC, Pop M (2013). Differential abundance analysis for microbial marker-gene surveys. Nat Methods.

[51] Breiman L (2001). Random forests. Mach Learn.

[52] Pedregosa F, Varoquax G, Gramfort A, Michel V, Thirion B, Grisel O (2011). Scikit-learn: machine learning in Python. J Mach Learn Res.

[53] McInnes P, Cutting M. Manual of procedures for human microbiome project: Core microbiome sampling, protocol A, HMP protocol no. 07–001, version 11. 2010. Current version: http://hmpdacc.org/doc/HMP_MOP_Version12_0_072910.pdf.

[54] Daniluk T, Tokajuk G (2006). Aerobic and anaerobic bacteria in subgingival and supragingival plaques of adult patients with periodontal disease. Adv Med Sci.

[55] Zijnge V, Van Leeuwen MBM, Degener JE, Abbas F, Thurnheer T, Gmür R (2010). Oral biofilm architecture on natural teeth. PLoS One.

[56] Aas JA, Paster BJ, Stokes LN, Olsen I, Dewhirst FE (2005). Defining the normal bacterial flora of the oral cavity defining the normal bacterial flora of the oral cavity. J Clin Microbiol.

[57] Kuczynski J, Liu Z, Lozupone C, McDonald D, Fierer N, Knight R (2010). Microbial community resemblance methods differ in their ability to detect biologically relevant patterns. Nat Methods.

[58] Xu Z, Malmer D, Langille MGI, Way SF, Knight R: Which is more important for classifying microbial communities: who’s there or what they can do? ISME J 2014;8:1–3. 10.1038/ismej.2014.157 PMC4260698 25171332

[59] Salim KY, De Azavedo JC, Bast DJ, Cvitkovitch DG (2007). Role for sagA and siaA in quorum sensing and iron regulation in Streptococcus pyogenes. Infect Immun.

[60] Bates CS, Montañez GE, Woods CR, Vincent RM, Eichenbaum Z (2003). Identification and characterization of a Streptococcus pyogenes operon involved in binding of hemoproteins and acquisition of iron. Infect Immun.

[61] Schymeinsky J, Mócsai A, Walzog B (2007). Neutrophil activation via beta2 integrins (CD11/CD18): molecular mechanisms and clinical implications. Thromb Haemost.

[62] Mahé F, Rognes T, Quince C, de Vargas C, Dunthorn M (2014). Swarm: robust and fast clustering method for amplicon-based studies. PeerJ.

[63] Tikhonov M, Leach RW, Wingreen NS (2015). Interpreting 16S metagenomic data without clustering to achieve sub-OTU resolution. ISME J.

