# Supplementary figures and images for: Phylogenetic approaches to microbial community classification
Source: Microbiome. 2015 Oct 5;3:47. doi: 10.1186/s40168-015-0114-5 (PMC4593236; doi:10.1186/s40168-015-0114-5)

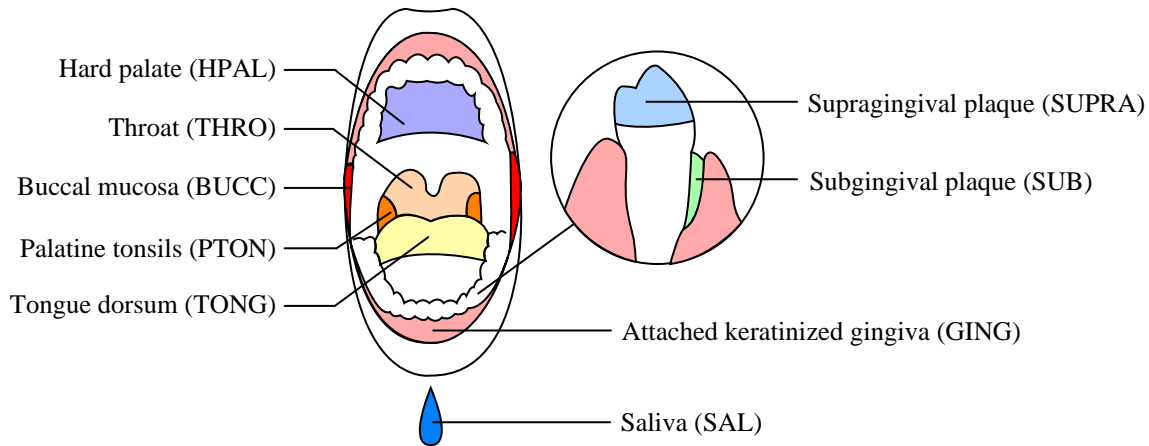

Supplement: Additional file 1: — Human oral cavity diagram drawn by SitePainter [ 35 ]. Different oral sites are identified using different colors, and abbreviations used in the text are given. (PDF 9 kb) [file 40168_2015_114_MOESM1_ESM.pdf]

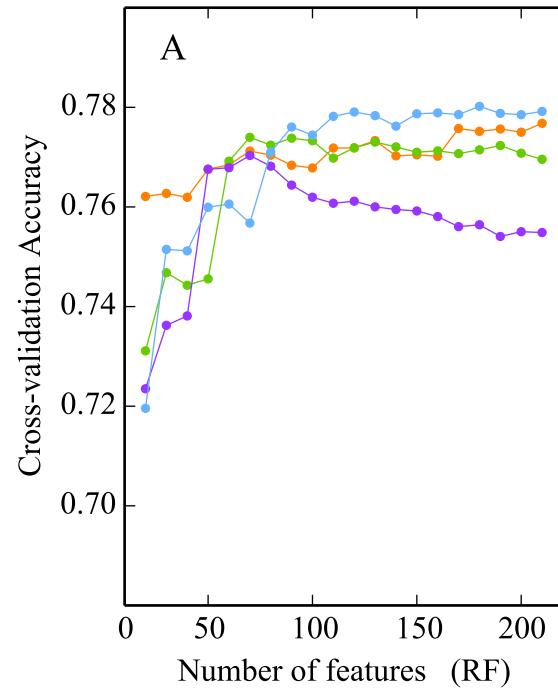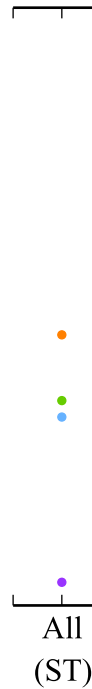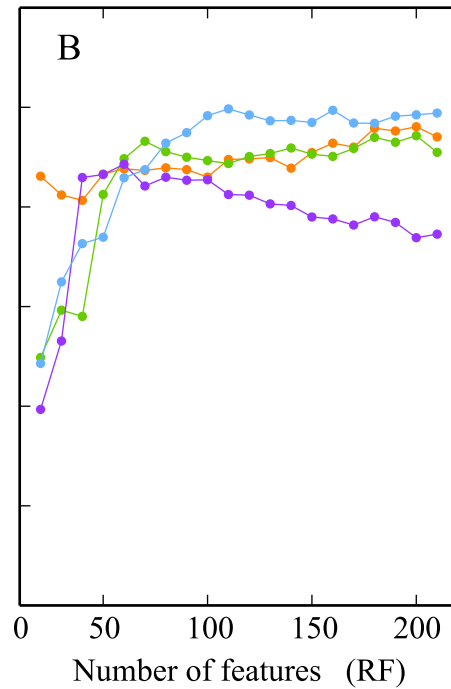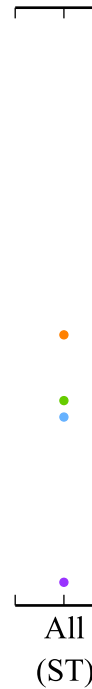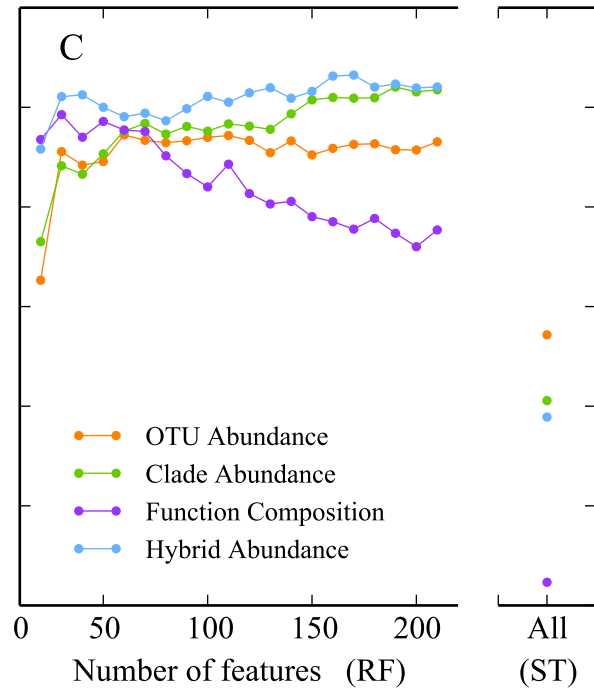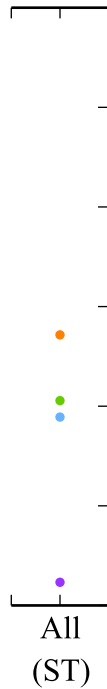

Supplement: Additional file 10: — Classification accuracy of RF and SourceTracker using different sets of input features. On the left side of each plot is the RF classification accuracy with sets of 10 to 200 of the top-ranked features according to information gain (A), chi-square (B), and RF feature permutation (C) criteria. The right-hand side of each plot shows Source Tracker’s classification accuracy with all features. The four types of input features used were OTUs only (orange markers); OTUs and clades (green markers); functional predictions made using PICRUSt (purple markers); and all generated features (blue markers). (PDF 28 kb) [file 40168_2015_114_MOESM10_ESM.pdf]

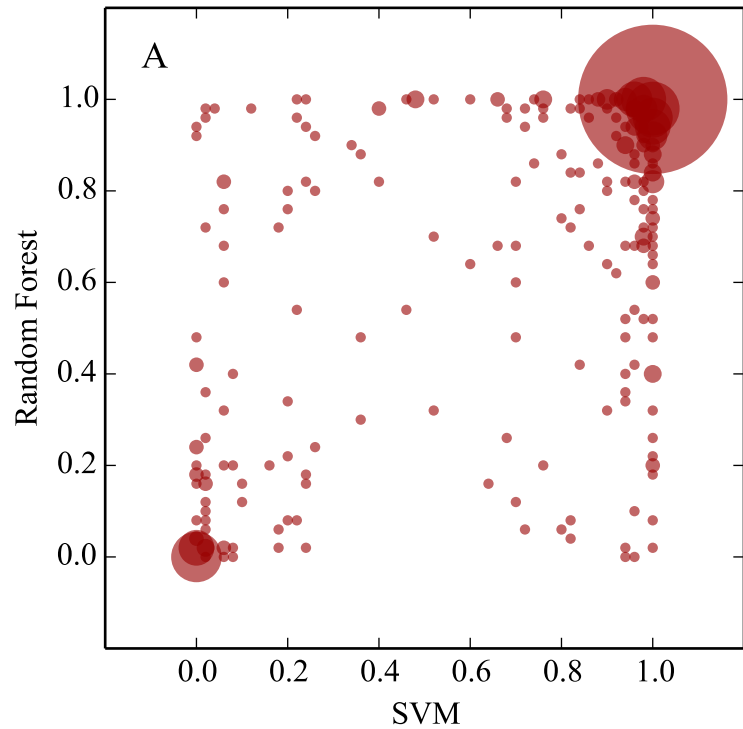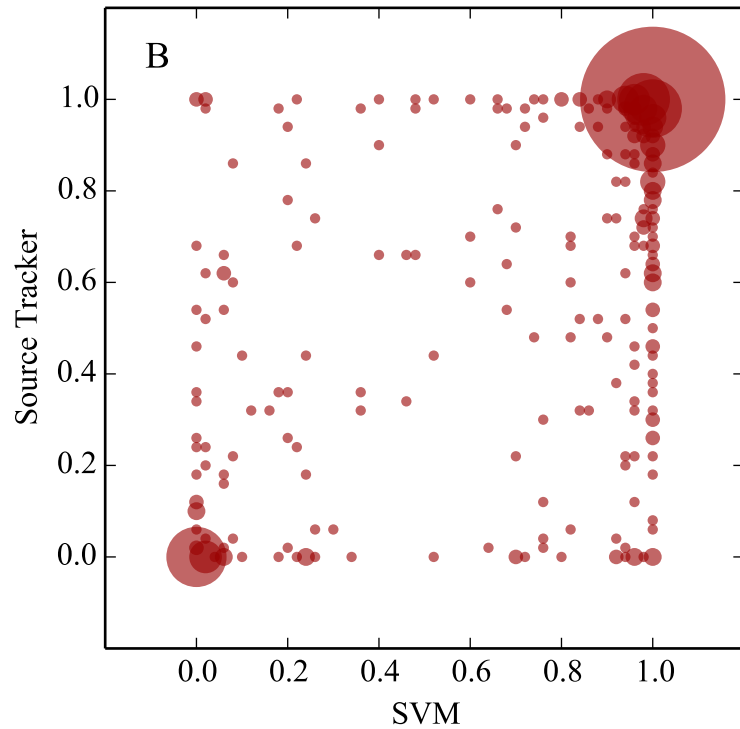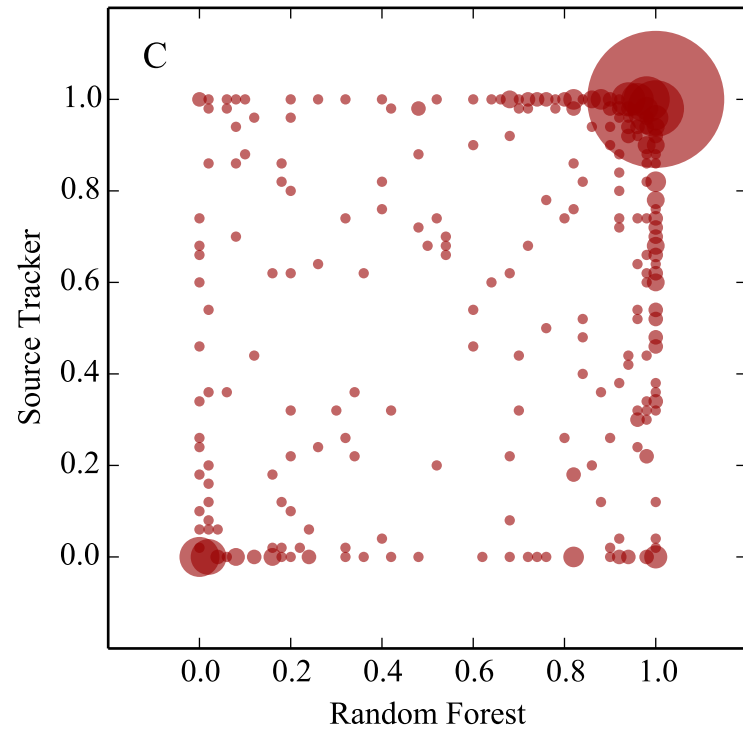

Supplement: Additional file 11: — The predictions on all samples: (a) SVM vs random forests, (b) SVM vs SourceTracker. The values on x- and y-axis indicate the frequency of samples that were correctly predicted by each of the two methods. The size of the nodes reflects the number of samples that were classified with the indicated accuracy, from 0 % by both classifiers in the lower left-hand corner to 100 % in the upper right-hand corner. (PDF 6,199kb) [file 40168_2015_114_MOESM11_ESM.pdf]
