# Supplementary material for: Phylogenetic approaches to microbial community classification
Source: Microbiome. 2015 Oct 5;3:47. doi: 10.1186/s40168-015-0114-5 (PMC4593236; doi:10.1186/s40168-015-0114-5)
Supplement: Additional file 4: — The performance of SVMs with different custom kernels. The distance metrics are ranked by their mean values and highlighted with colors consistent to the similarity-based clustering of [5]. Highly correlated measures are grouped in one color set. (PDF 7 kb) [file 40168_2015_114_MOESM4_ESM.pdf]

| ***distance_measures*** | ***otu_count*** | ***otu_abundance*** | ***rarefication*** | ***css_normalization*** | ***mean ±s.d*** |
|---|---|---|---|---|---|
| Canberra | 0.7623 | 0.7623 | 0.7662 | 0.769 | 0.765 ±0.003 |
| Kulczynski | 0.7591 | 0.7574 | 0.7662 | 0.769 | 0.763 ±0.005 |
| Bray-Curtis | 0.7574 | 0.7574 | 0.7662 | 0.769 | 0.763 ±0.005 |
| Hellinger | 0.7558 | 0.7556 | 0.7728 | 0.7624 | 0.762 ±0.007 |
| Soergel | 0.7591 | 0.7541 | 0.7562 | 0.7657 | 0.759 ±0.004 |
| Pearson | 0.7426 | 0.7426 | 0.7512 | 0.7673 | 0.751 ±0.010 |
| Morisita-Horn | 0.7426 | 0.7426 | 0.7496 | 0.7673 | 0.751 ±0.010 |
| unweighted UniFrac | 0.7294 | 0.7459 | 0.7695 | 0.7294 | 0.744 ±0.016 |
| Chi-square | 0.7442 | 0.7409 | 0.7363 | 0.7393 | 0.740 ±0.003 |
| Normalized weighted UniFrac | 0.7277 | 0.7327 | 0.7512 | 0.7376 | 0.737 ±0.009 |
| weighted UniFrac | 0.7343 | 0.736 | 0.7396 | 0.7376 | 0.737 ±0.002 |
| Gower | 0.7393 | 0.7508 | 0.6617 | 0.7558 | 0.727 ±0.038 |
| Manhattan | 0.7079 | 0.7277 | 0.6202 | 0.7591 | 0.704 ±0.052 |
| Euclidean | 0.6947 | 0.736 | 0.6219 | 0.7541 | 0.702 ±0.051 |
| mean ±s.d. | 0.740 ±0.019 | 0.746 ±0.010 | 0.731 ±0.052 | 0.756 ±0.014 | / |
